# Impact of modeled field of view in electroconvulsive therapy current flow simulations

**DOI:** 10.3389/fpsyt.2023.1168672

**Published:** 2023-05-18

**Authors:** Alexander Guillen, Christopher C. Abbott, Zhi-De Deng, Yu Huang, Paula Pascoal-Faria, Dennis Q. Truong, Abhishek Datta

**Affiliations:** ^1^Research and Development, Soterix Medical, Woodbridge, NJ, United States; ^2^Department of Psychiatry, University of New Mexico, Albuquerque, NM, United States; ^3^Noninvasive Neuromodulation Unit, Experimental Therapeutics and Pathophysiology Branch, National Institute of Mental Health, National Institute of Health, Bethesda, MD, United States; ^4^Department of Mathematics ESTG and CDRSP Polytechnic Institute of Leiria, Leiria, Portugal; ^5^City College of New York, New York, NY, United States

**Keywords:** ECT, electroconvulsive therapy, extent, human head model, validation, simulation, MRI

## Abstract

**Background:**

The field of view (FOV) considered in MRI-guided forward models of electroconvulsive therapy (ECT) are, as expected, limited to the MRI volume collected. Therefore, there is variation in model extent considered across simulation efforts. This study examines the impact of FOV on the induced electric field (E-field) due to two common electrode placements: right unilateral (RUL) and bilateral (BL).

**Methods:**

A full-body dataset was obtained and processed for modeling relevant to ECT physics. Multiple extents were derived by truncating from the head down to four levels: upper head (whole-brain), full head, neck, and torso. All relevant stimulation and focality metrics were determined. The differences in the 99th percentile peak of stimulation strength in the brain between each extent to the full-body (reference) model were considered as the relative error (RE). We also determine the FOV beyond which the difference to a full-body model would be negligible.

**Results:**

The 2D and 3D spatial plots revealed anticipated results in line with prior efforts. The RE for BL upper head was ~50% reducing to ~2% for the neck FOV. The RE for RUL upper head was ~5% reducing to subpercentage (0.28%) for the full-head FOV. As shown previously, BL was found to stimulate a larger brain volume—but restricted to the upper head and for amplitude up to ~480 mA. To some extent, RUL stimulated a larger volume. The RUL-induced volume was larger even when considering the neural activation threshold corresponding to brief pulse BL if ECT amplitude was >270 mA. This finding is explained by the BL-induced current loss through the inferior regions as more FOV is considered. Our result is a departure from prior efforts and raises questions about the focality metric as defined and/or inter-individual differences.

**Conclusion:**

Our findings highlight that BL is impacted more than RUL with respect to FOV. It is imperative to collect full-head data at a minimum for any BL simulation and possibly more. Clinical practice resorts to using BL ECT when RUL is unsuccessful. However, the notion that BL is more efficacious on the premise of stimulating more brain volume needs to be revisited.

## 1. Introduction

Severe psychiatric disorders (e.g., clinical depression) are among the main public health concerns worldwide. In the United States alone (US), the National Institute of Mental Health reports that major depressive disorder affects approximately 17.3 million American adults in any given year, or 7.1% of the population 18 and older (1), many of whom are treatment resistant. Clinical depression co-occurs with other illnesses and medical conditions, such as heart attack and diabetes, which are among the leading causes of death in the United States (2). Treatment for depression includes antidepressants, psychotherapy, and electroconvulsive therapy (ECT) to treat severe, life-threatening cases that have not responded to medications.

Electroconvulsive therapy induces a generalized seizure in anesthetized patients by delivering an electrical current to the brain through electrodes on the scalp (3). Studies have shown that ECT is more effective than drug therapy, with 50–60% of patients achieving rapid relief from depression after a course of ECT, compared to 10–40% with pharmacotherapy or psychotherapy (4). ECT is also associated with low rates of psychiatric hospitalizations and a rapid reduction in suicide drive among psychiatric patients (5). Despite its high effectiveness in clinical use, ECT use is low [it is administered to an estimated 100,000 people annually in the United States (6)], as it is stigmatized due to the risk of cognitive side effects.

Advances in ECT administration are then focused on reducing its adverse effects on patients. Sackeim et al. found that high-dose right unilateral (RUL) ECT electrode configuration was as effective as a robust form of bilateral (BL) ECT, resulting in less severe and persistent cognitive impairment (7). Other studies have pointed out the importance to understand the ECT stimulus parameters and the electric field (E-field) characteristics in order to provide a more focal stimulation—closer to the neural firing threshold and thus reduce unnecessary ECT intensity [800–900 mA (4)] for seizure induction that may put patients at higher risk for adverse events.

Modern-day ECT parameter selection consists of electrode placement choice (RUL or BL) followed by corresponding values for pulse width, frequency, pulse train duration, and amplitude (26–29). The ECT clinician picks starting electrode placement and pulse width prior to treatment. For frequency and pulse train duration, either demographic variables (age and sex) or seizure titration determines values for the individual patient. Finally, a fixed amplitude (800 or 900 mA) is chosen based on the available commercial ECT device. In some treatment centers, the ECT course is initiated with parameters that are associated with less cognitive risk (ultra-brief pulse width of 0.3 ms and RUL) and if insufficient for seizure induction or underwhelming clinical efficacy after the first few treatment sessions, switching to more efficacious parameters (brief pulse width and BL). This empirical approach is followed to ensure that the ECT patient ultimately receives the adequate efficacy dose.

Computational models of current flow continue to add meaningful value to several electrical stimulation modalities for retrospective analysis (e.g., interpret clinical outcome) and for prospective ones (stimulation parameter planning and optimization). For example, forward models that are used for deep brain stimulation (DBS) lead selection are now clinically approved and part of routine care (8). In ECT, computational models have helped quantify stimulation strength and focality (3, 9–11), explored the influence of white matter conductivity (12), field inside fetal brain during pregnancy (13), related outcomes (14), etc. Since these models are directly derived from a structural MRI scan, there is variability in extent (or the dataset's axial range) considered, due to a lack of standardization.

This study was primarily motivated to inform the extent/field of view (FOV) beyond which additional MRI data collection becomes unnecessary for accurate prediction of ECT-induced stimulation metrics. Previous efforts indicate RUL is generally more focal (i.e., stimulating a lower percentage of brain volume) than BL (3). Clinical ECT studies corroborate the same indirectly, and as mentioned above, typically start a course with an RUL montage. Therefore, we were interested in exploring how the incorporation of various extents affects the two conventional electrode placements and whether there are montage-specific considerations. Given the temporal placement of both electrodes in BL ECT, one can hypothesize a greater impact than RUL on the extent considered. Furthermore, prior modeling efforts have considered a common value for neural activation threshold when comparing montages. However, since ultra-brief RUL is switched to brief BL in real-world practice, the comparison should also be made with respect to the updated neural activation threshold value accounting for the wider pulse width. In this study, we first developed an anatomically realistic finite element model of a full body. Four additional versions of the model were considered by truncating the geometry to different extents (upper head, full head, neck, and torso). We simulated ECT-induced current flow and computed all typical stimulation and focality metrics. We finally reported on the impact of modeled FOV and propose the extent required for accurate current flow prediction.

## 2. Methods

The simulation of the E-field distribution was performed as follows:

### 2.1. Anatomical dataset and model geometry

The Caucasian brain atlas (ICBM-152) was obtained from the Montreal Neurological Institute (MNI, Montreal, Canada) (15). We considered a representative average template appropriate given the goal to shed light on the impact of FOV that should hold across a large population. The impact of individual effects was not the focus of this study. With regard to the whole-body dataset, the “Duke” data was obtained from the Virtual Population (ViP) family (16), which is a set of detailed high-resolution anatomical models created from magnetic resonance image data of volunteers.

### 2.2. Image processing and segmentation

**Figure 1:**
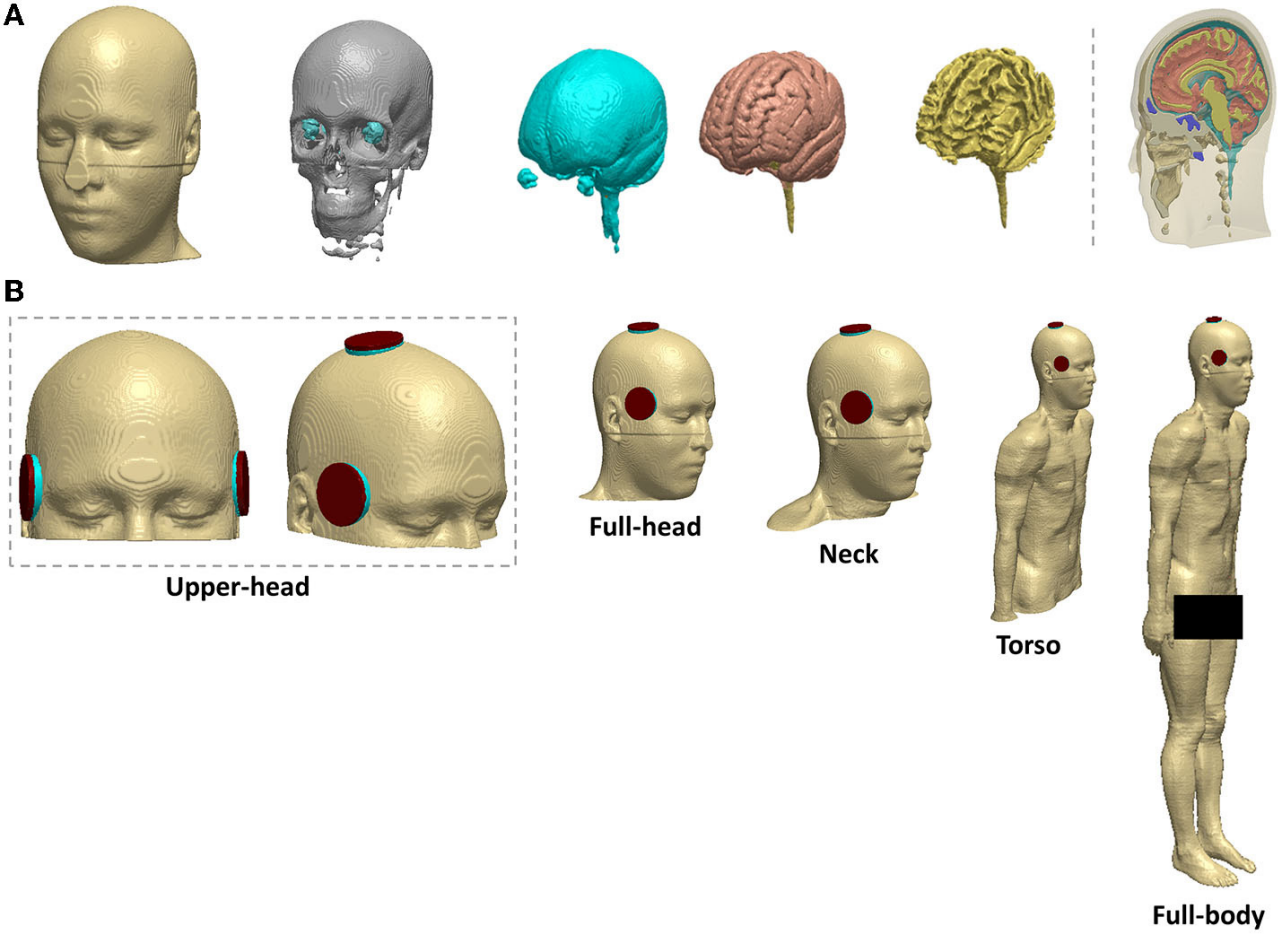
Head segmentation, electrode placement, and different field of view (FOV) considered **(A)** segmentation of the MNI 152 template into tissue categories **(B)** BL and RUL placement. Varying FOV considered from left to right: upper head, full-head, neck, torso, and full-body (reference).

Segmentation of the MRI data into tissue categories (Figure 1), such as skin and skull, was based on an extensive prior study by our group (17–20). Using the software Simpleware (Synopsys Ltd., CA, United States), the segmented MNI 152 dataset was modified to establish a continuous cerebrospinal fluid (CSF) and fused with the body of the Duke human model (Figure 1). Simpleware was also used to design the stimulation electrodes and integrate them with the whole-body model, and generate the FEM meshes with a high-quality factor.

### 2.3. Electrode placement and model computation

The ECT electrode configurations were right unilateral (RUL) and bilateral (BL) (Figure 1). The electrodes and gel compartments were modeled according to actual ECT administration. For BL ECT, the center of both electrodes was placed bilaterally at the frontotemporal positions of the head, 2.5 cm above the midpoint of an imaginary line connecting the outer canthus of the eye and the tragus of the ear (3). In RUL ECT, one electrode was placed 2.5 cm to the right of the vertex of the head, and the second electrode was centered at the right frontotemporal location like the BL ECT configuration. The modeled ECT electrodes were circular with a radius of 25 mm. The shape of the gel/paste was also circular with a radius equal to that of the electrodes.

As shown in Figure 1, the resulting model was then truncated from the head down into four distinct sections: upper head, full head, neck, and torso. In total, 10 different volumetric meshes were generated and imported separately into COMSOL Multiphysics 5.6 (COMSOL Inc., MA, USA) to obtain E-field surface plots of the brain—selecting the space dimension as 3D, the physics as electric current (ec), and study type as stationary. The isotropic and homogeneous electrical conductivity values assigned to each comportment in (S/m) were as follows skin: 0.465; bone: 0.01; CSF: 1.65; gray matter: 0.276; white matter: 0.126; muscle: 0.35; male reproductive system: 0.232; urinary bladder wall: 0.408; intestines: 0.164; cartilage: 1.01; liver: 0.221; kidney: 0.403; air: 1e^−15^; sponge: 1.4; and electrode: 5.9 e^7^ (18, 21, 22). The relative permittivity remained constant for all compartments as the model was solved as a quasi-static system. Finally, the relevant boundary conditions were then imposed: normal current intensity at the anode electrode; ground condition at the cathode electrode; and all other external surfaces treated as insulated (17).

### 2.4. Data analysis

For the data analysis, we analyzed the spatial distribution of the E-field induced in the brain by ECT, characterized by 3D cortical and 2D cross-sectional surface plots. These images depict the stimulation strength relative to the neural activation threshold by dividing the E-field by the E-field threshold (E/E_th_). We considered the previously estimated neural activation E-field threshold for ultra-brief pulse ECT (0.25 V/cm) by Deng et al. (9). To fully delve into the impact of FOV considered, we calculated the 99th percentile peak of the induced stimulation strength in the entire brain relative to E_th_ as ECT amplitude is scaled (100–900 mA) (Table 1). If one were to consider the full-body model as the ground truth as it reflects the actual experimental condition, we introduce a relative error (RE) metric to systematically quantify the impact of FOV.


$$\begin{array}{l} RE=\left(M_{X}-M_{FB}\right)/M_{FB}*100\% \end{array}$$

**Table 1:** 99th percentile peak of the stimulation strength (E-field magnitude relative to a neural activation threshold, E/E_th_) in the brain at 100–900 mA using the RUL and BL ECT configuration, respectively.

**ECT Dose (RUL)**	**Upper-head**	**Full-head**	**Neck**	**Torso**	**Full-body**
100mA	0.9803	0.9388	0.9380	0.9372	0.9361
200mA	1.9607	1.8776	1.8760	1.8744	1.8722
300mA	2.9410	2.8164	2.8140	2.8116	2.8083
400mA	3.9214	3.7552	3.7520	3.7489	3.7444
500mA	4.9017	4.6940	4.6900	4.6861	4.6805
600mA	5.8821	5.6328	5.6280	5.6233	5.6166
700mA	6.8624	6.5716	6.5660	6.5605	6.5527
800mA	7.8427	7.5104	7.5040	7.4977	7.4888
900mA	8.8231	8.4492	8.4420	8.4349	8.4249
**ECT Dose (BL)**	**Upper-head**	**Full-head**	**Neck**	**Torso**	**Full-body**
100mA	1.3911	0.9516	0.9493	0.9314	0.9299
200mA	2.7821	1.9033	1.8987	1.8628	1.8598
300mA	4.1732	2.8549	2.8480	2.7942	2.7898
400mA	5.5642	3.8066	3.7973	3.7257	3.7197
500mA	6.9553	4.7582	4.7467	4.6569	4.6496
600mA	8.3463	5.7099	5.6960	5.5884	5.5795
700mA	9.7374	6.6615	6.6453	6.5198	6.5094
800mA	11.1284	7.6132	7.5947	7.4511	7.4394
900mA	12.5195	8.5648	8.5440	8.3827	8.3693

Where, **M**_**x**_: Model X; X being the FOV under consideration; and **M**_**FB**_: Model (full body).

We further compared the ratio of the right to left hemisphere median E-field magnitude across all FOV extents to quantify lateralization. The stimulation focality of each condition by virtue of the percentage of brain volume exposed to the E-field that is stronger than the neural and robust activation threshold was also determined. Additionally, the brain volume percentages with respect to brief pulse ECT were calculated. The robust activation threshold was assumed to be 1.4 ^*^ E_th_ based on prior studies (23).

## 3. Results

### 3.1. Cortical surface and cross-section E-field magnitude

**Figure 2:**
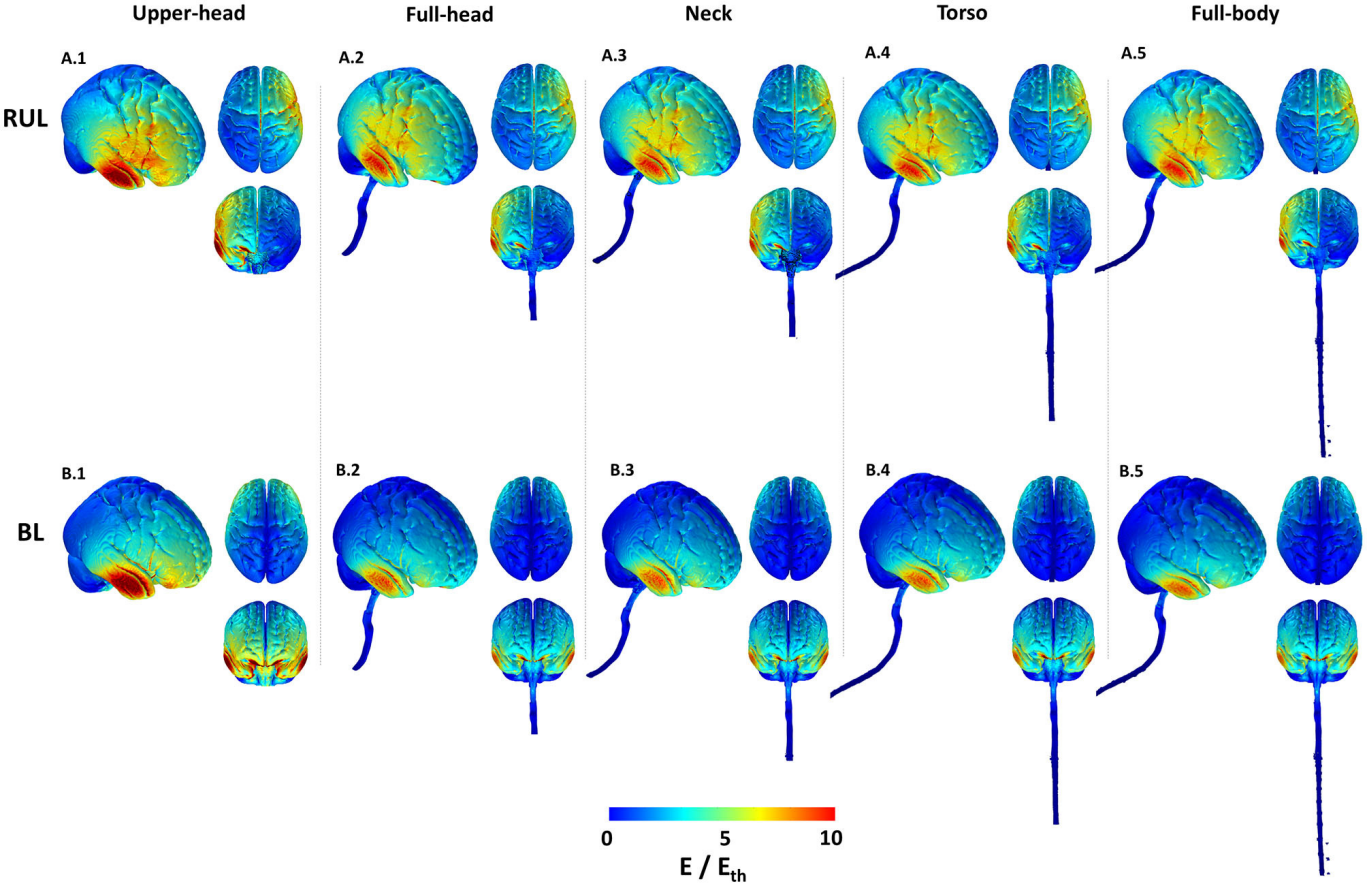
Cortical 3D surface E-field magnitude plots of the RUL and BL electrode configurations across all extents. The images indicate E-field strength relative to the neural activation threshold (E_th_ = 0.25 V/cm). The images are plotted to the same scale to highlight differences. For each combination, the right lateral view along with the top and anterior views are shown.

The cortical 3D surface plots show that the E-field distributions differ considerably between the stimulated montages, consistent with previous studies (3, 11). For the RUL montage, the dominant current flow extends between the two stimulation electrodes (i.e., around the vertex and the frontotemporal location). The overall current spread is however wide and diffuse, with flow extending from the right frontal to the right posterior regions (Figure 2). Also, one may perceive from the top and anterior views in Figure 2 that the E-field for the RUL configuration is relatively more spatially distributed in the superior regions, while the BL montage tends to favor current flow through the anterior and inferior regions of the brain. Although the current flow extends to lower regions of the brain using both electrode montages, based on the anterior and top views, it is evident that the current flow of the BL montage is diffused further down extending into the pons, medulla oblongata, and the beginning of the spinal cord.

For both montages, the upper head model resulted in the highest stimulation strength (E-field magnitude relative to the neural activation threshold) in comparison to the other extents. The induced stimulation strength thereafter (i.e., as more FOV is considered) did not change noticeably. Both montages indicate increased current flow/concentration in the temporal region [directly underneath the temporal electrode(s)] reflecting thinner bone in the region.

**Figure 3:**
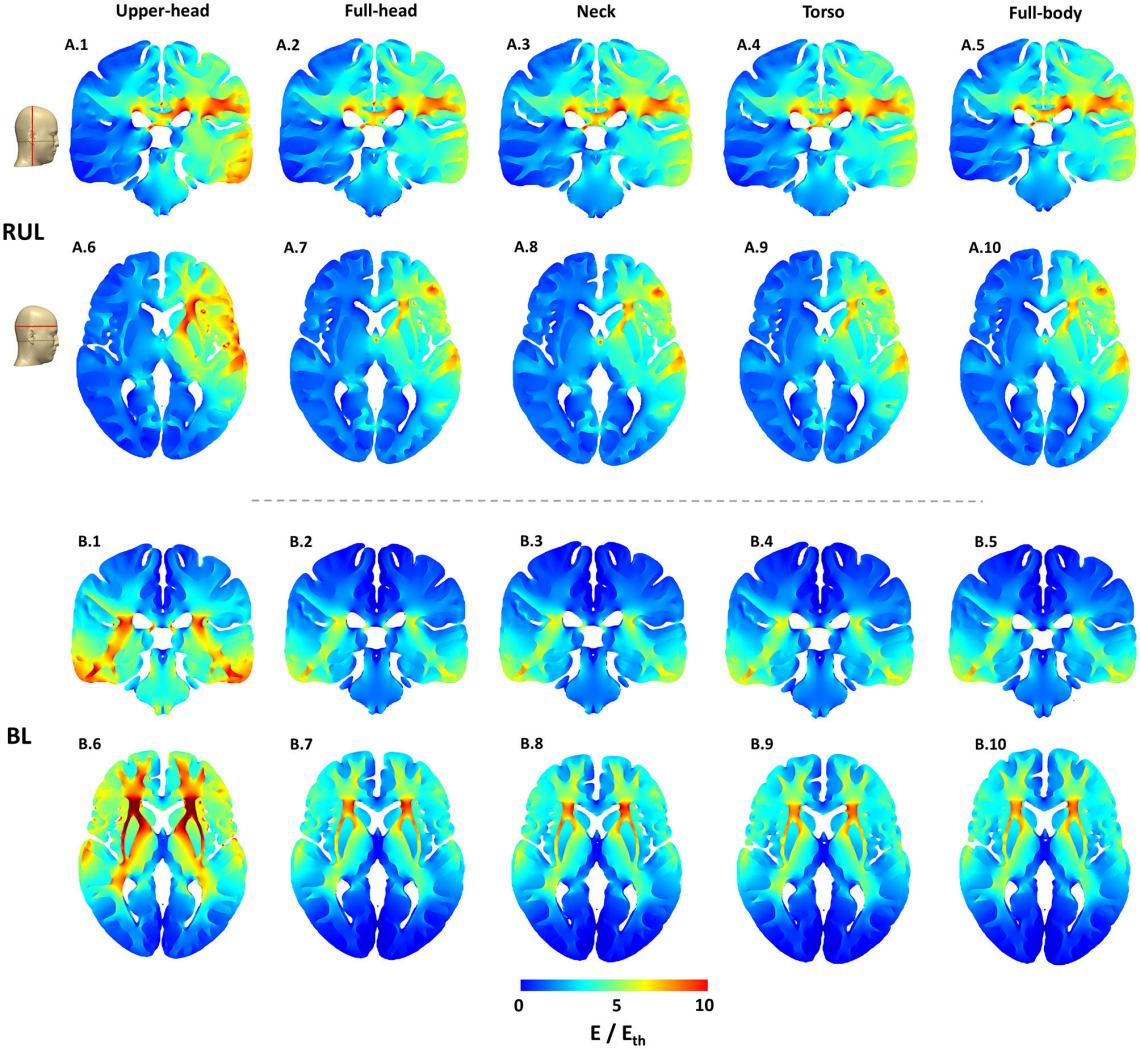
Cortical cross-section 2D stimulation strength (E/E_th_) plots for both montages across all extents considered. The exact location of the representative slices is indicated by the red lines on the scalp mask. The color scale is identical to Figure 2.

The 2D cross-sectional plots in Figure 3 help examine depth focality/flow in the deeper subcortical regions. These images further solidify some of the earlier observations based on the 3D surface plots. For instance, for RUL, flow extends from the head apex ending at the temporal location in the right hemisphere (see coronal image) and flow extends from the right frontal to the right posterior regions (see the axial image). For BL, the posterior regions are “spared” in comparison and the flow is concentrated in the inferior regions. This observation is more evident in the extent starting from the full-head to models with more FOV. Owing to the shortest FOV in the upper head model, current cannot flow any inferior to the geometry/tissue available, thereby resulting in the highest stimulation strength to other extents. Furthermore, the BL montage results in higher stimulation strength than the RUL montage, as clearly evident by the axial slice for the upper head montage (Figure 3.B.6). However, as more FOV is considered, induced stimulation strengths look qualitatively similar.

While the RUL montage is clearly lateralized, there is some current flow into the left hemisphere. The symmetricity of current flow in the BL montage which reflects the electrode placement is clearly demonstrated by both the coronal and the axial images. Finally, for both montages and similar to the 3D surface plots, there is a substantial drop-off in stimulation strength going from the upper head to the full-head FOV and then not changing much thereafter.

**Figure 4:**
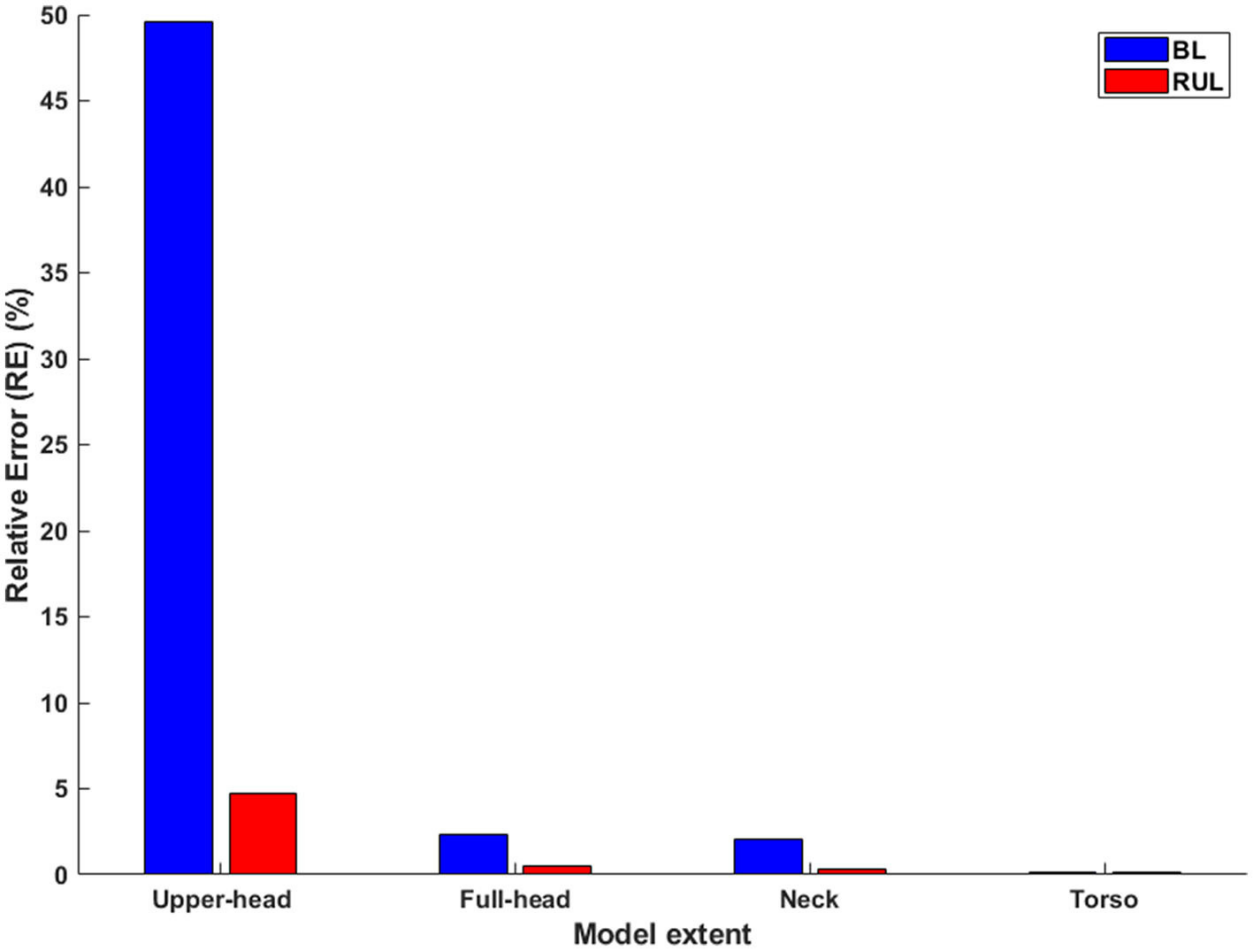
The relative error in relation to the full-body model (Left: BL; Right: RUL) across all extents. Considering the whole-body model as the reference model, the relative error is calculated for each of the remaining extents.

For both montages, overall, it is clear to observe drops in induced values at every transition, as more and more FOV is considered (Table 1 and Figure 4). The transition from the upper head to the full-head results in the biggest drop than the other transitions. For RUL and ECT amplitude of 100 mA, RE between the upper head and the full-body model was 4.72%. The relationship between ECT current intensity and E/E_th_ is expected to be linear and is readily confirmed by considering RE between the montages at a different current intensity. The corresponding RE between the full-head, neck, and torso models to the full-body model are 0.28, 0.20, and 0.11%, respectively. For the BL amplitude of 100 mA, RE between the upper head and the full-body model was 49.59%—confirming that BL is much more substantially impacted than RUL by the limited FOV. The corresponding RE between the full-head, neck, and torso models to the full-body model are 2.33, 2.08, and 0.16%, respectively.

### 3.2. Laterality of stimulation

**Figure 5:**
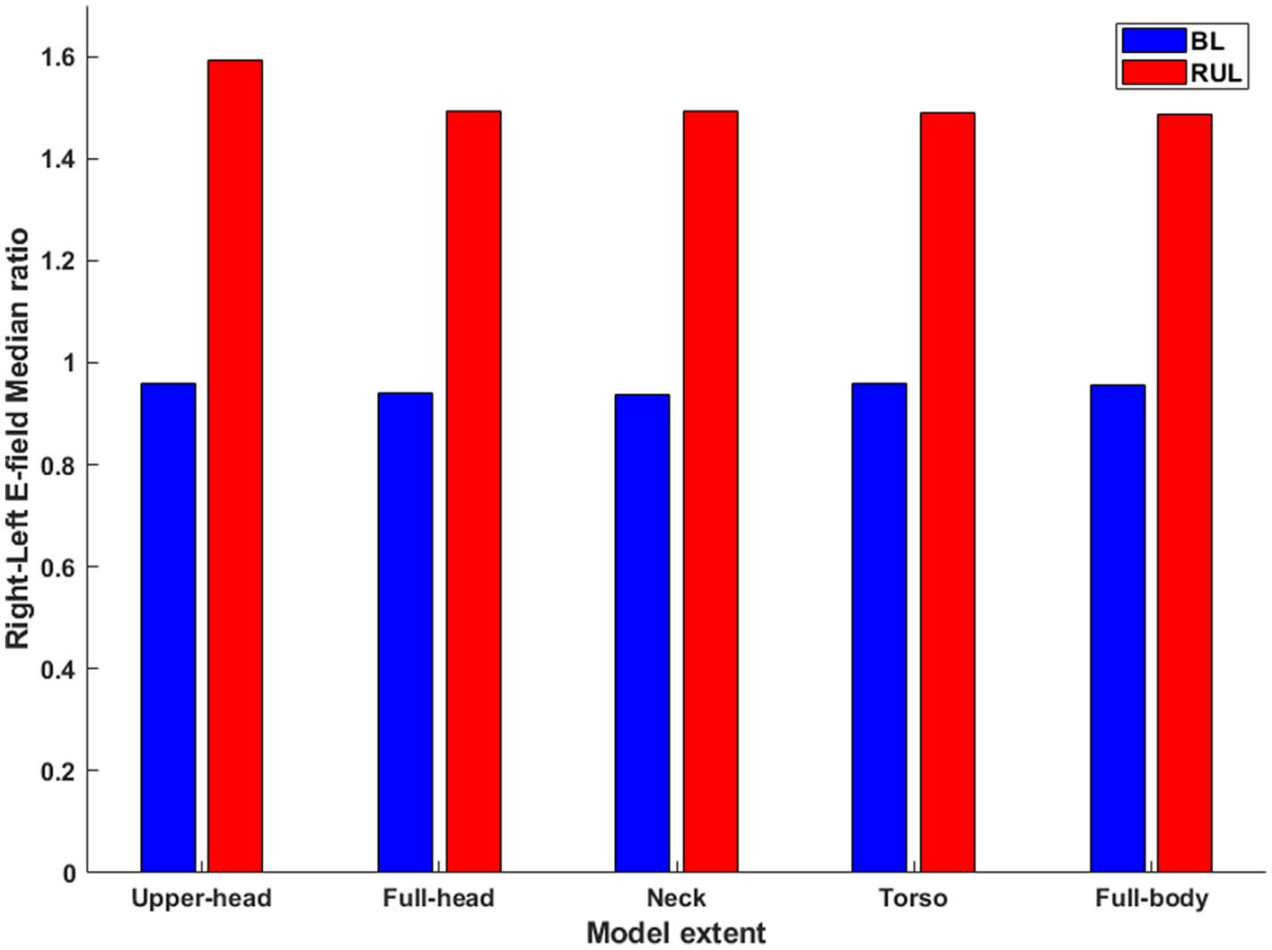
The ratio of the median E-field magnitude in the right hemisphere relative to the left hemisphere. All combinations of montage (RUL and BL) and extents considered.

The laterality of stimulation was quantified by determining the ratio of the median E-field magnitude of the right to the left hemisphere (Figure 5). As expected (3), the ratio for the lateralized RUL electrode configuration is higher than the BL electrode configuration to all extents considered. There is negligible impact of different head extents on the laterality ratios for the BL montage with all extents indicating a value ~1. This is expected given the perfectly symmetrical nature of the montage. The ratio of the upper head model for RUL is the highest (1.59) explained by more current flow into the right hemisphere due to the reduced FOV (see Figure 3.A.1). The ratio drops to 1.49 for the full-head model and stays largely unchanged for additional head extents considered. The drop is explained by additional current flow into the left hemisphere (or reduced current flow into the right hemisphere) as more FOV is considered. Whereas, for the BL montage, owing to the symmetric design, the extent considered has virtually no impact on the laterality ratios—as one can expect.

### 3.3. Stimulation focality (brain volume percentage above neural activation threshold)

**Figure 6:**
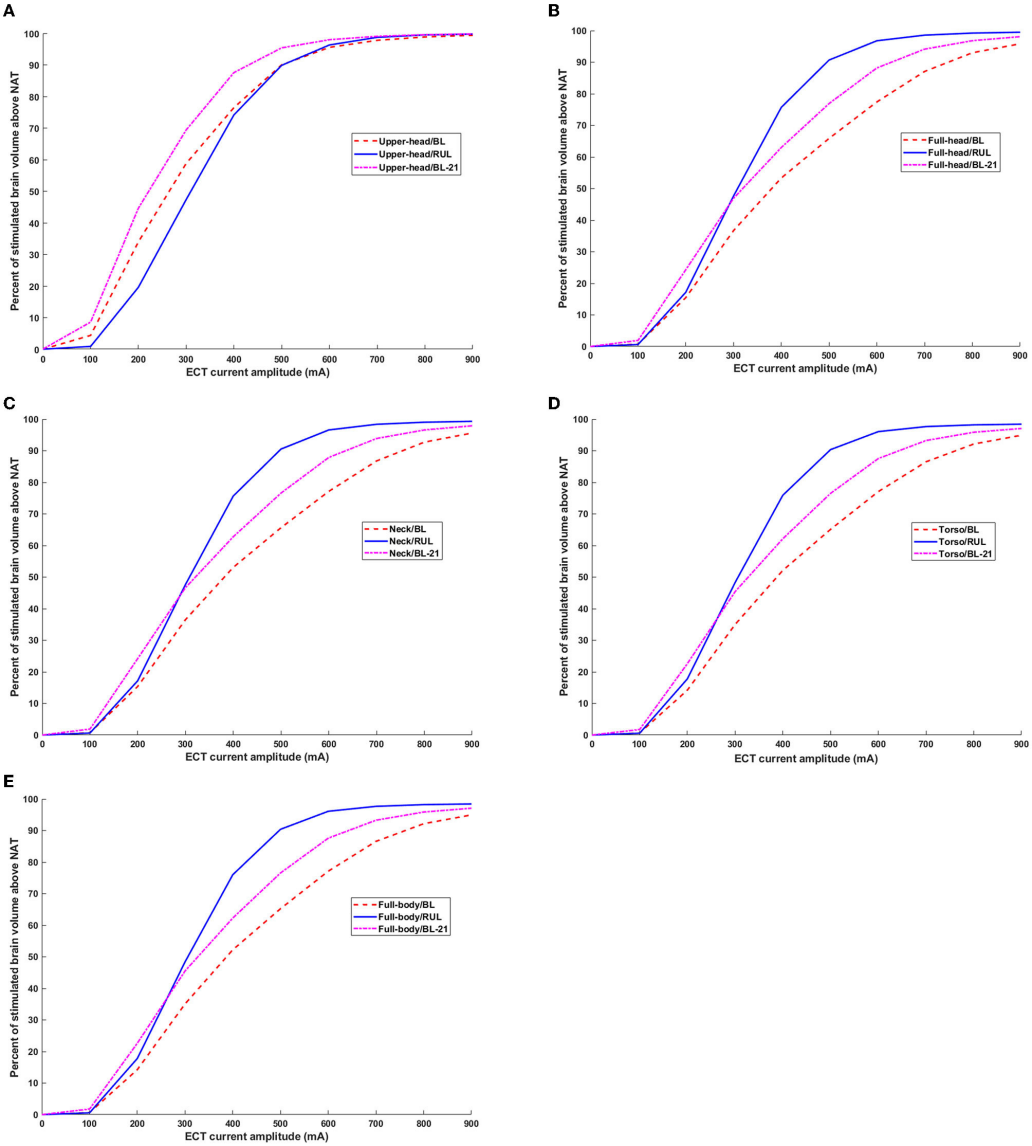
Percentage of brain volume stimulated above neural activation threshold as a function of ECT current amplitude. Both thresholds corresponding to ultra-brief and brief pulse are considered. BL_21_ refers to the trace corresponding to BL montage when using E_th_ of 21 V/m. **(A–E)** Upper head, full-head, neck, torso, and full-body.

Plotting the ECT current amplitude vs. the stimulated brain volume percentage, the relationship between these parameters is a combination of both linear and non-linear phases (Figure 6). For the upper head FOV, the percentage of the brain volume stimulated above E_th_ by the BL configuration is clearly higher than the RUL montage for ECT amplitude up to ~480 mA (Figure 6A). Thereafter, both BL and RUL induced curves merge, and both reach maximum percentage at ~700 mA and higher. When considering E_th_ of 21 V/m (i.e., E_th_ corresponding to brief pulse BL), BL_21_ was found to stimulate a higher brain volume than RUL for almost the entire ECT amplitude range until both curves align and saturate. When considering the full-head extent, RUL initially starts off similar to BL but lower than BL_21_ (Figure 6B). At ECT amplitudes ~210 mA and ~290 mA, RUL surpasses BL and BL_21_, respectively.

For the remaining extents besides the full-body model, during the initial ECT doses (i.e., until ~210 mA), both RUL and BL induce a similar percentage of brain volume stimulation but only when considering brief pulses. For the full-body model, RUL starts off higher than BL even during initial ECT doses (Figure 6E). Starting with the full-head extent and until the full-body model, RUL surpasses BL_21_ ~290 mA and gets to the maximum percentage ~800 mA. Whereas, for both BL pulses, the relationship between the ECT amplitude and stimulated brain volume percentage is more or less linear for the range considered.

It is, thus, important to note that irrespective of the extent, for RUL montage, the stimulated brain volume curves saturate (i.e., reach 100%) at high ECT doses (~700-900 mA), producing negligible changes thereafter. For all extents, the stimulation focality values for the RUL montage resemble a sigmoid function. Whereas, for the BL montage (for both ultra-brief and brief pulses), the relationship between ECT amplitude and brain volume stimulated is largely linear besides the upper head model.

### 3.4. Stimulation focality: comparison of neural and robust neural activation threshold

**Figure 7:**
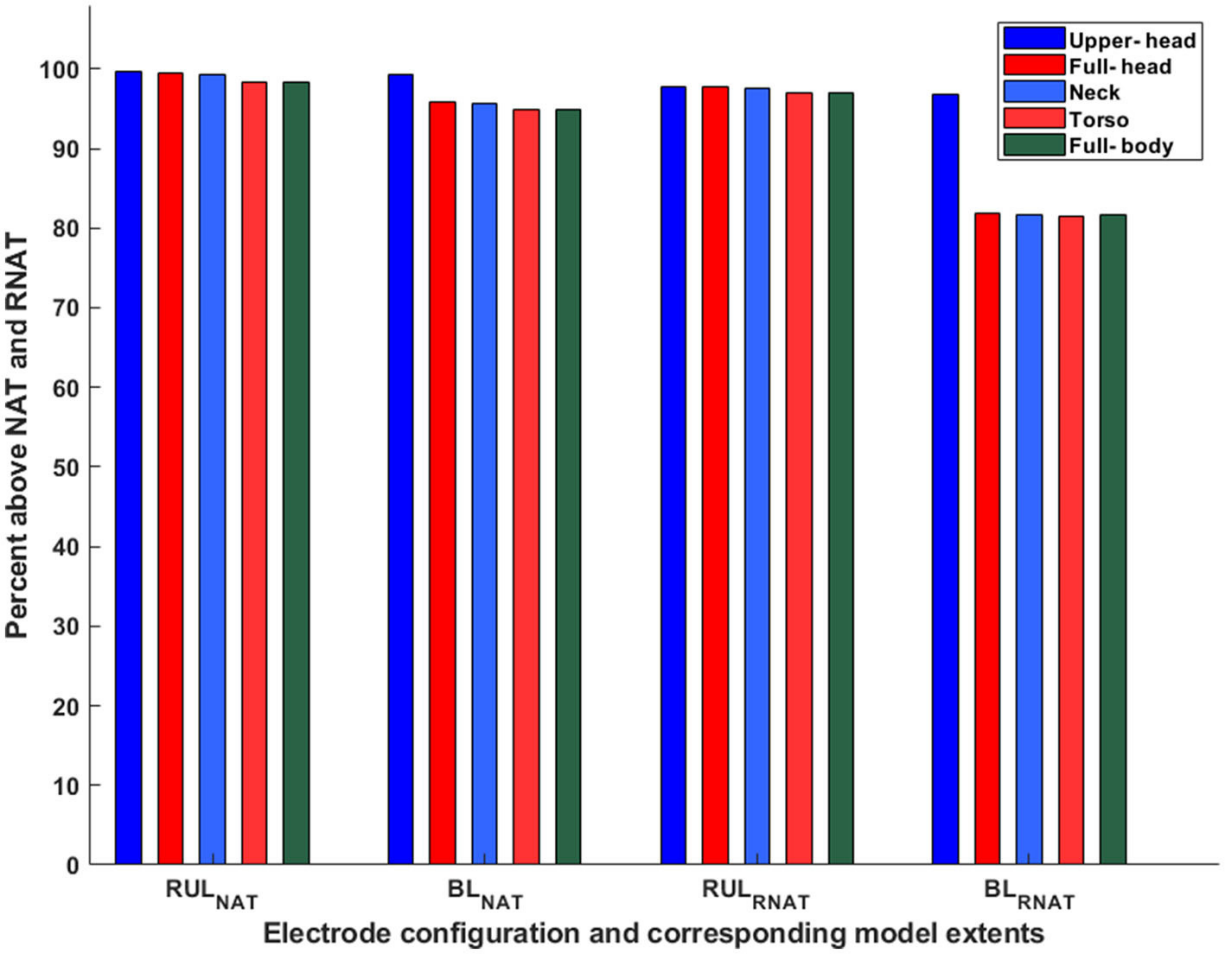
Percentage of brain volume stimulated above neural and robust neural activation threshold. The subscripts NAT and RNAT denote the neural activation threshold and the robust neural activation threshold, respectively. E_th_ corresponds to ultra-brief pulses (25 V/m).

Finally, we estimated the overall focality of stimulation with respect to the robust neural activation threshold (E ≥ 1.4^*^E_th_) (Figure 7). For the sake of simplicity, we only considered the E_th_ corresponding to ultra-brief BL and RUL pulses (25 V/m). Overall, as expected, the results observed with the neural activation threshold are also consistent with the robust neural activation threshold, as the focality metric used is determined relative to a scaling factor of 1.4. Specifically, RUL is *less sensitive* to the FOV considered with the marginal change going from the upper head to the full head and then no change thereafter. The BL montage, owing to the two temporal electrodes, indicates a noticeable change going from the upper head to the full head. There is no marked change thereafter as more FOV is considered. When comparing montages, these plots confirm that overall, the RUL montage stimulates a higher percentage of the brain volume.

## 4. Discussion

This study investigated the impact of the modeled field of view on ECT current flow computation for the first time. The variation in typical ECT stimulation/focality metrics as FOV is increased from the upper head to full body over five extents was considered.

Our results demonstrate that the modeled FOV impacts induced stimulation metrics. The level of impact is as expected, a function of the montage considered and its overall expected current flow pattern. Given the flow in the inferior regions due to BL, there is a profound impact of considering model FOV limited to the level of the base of the full brain (upper head). However, the relative error falls quickly to ~2% when considering the neck, ultimately reaching a subpercentage error at the torso FOV. For the RUL montage, as the relative error to the ground truth is already at subpercentage (0.28%) at the full-head extent, we do not recommend collecting any more data beyond this extent. For the BL montage, consideration of FOV extending to full head is imperative. Further extent consideration would depend on the goal of the modeling process. For instance, a ~2% error may not be meaningful when comparing stimulation outcomes across multiple subjects that all used the same BL montage. However, for a modeling validation exercise (21), further reduction may be critical to ensure matching predictions to recordings.

Our results indicate that contrary to previous reports, the model used in this study showed that the RUL montage is less focused than the BL montage. This finding is explained by the consideration of different FOVs in this study, thereby indicating its utility. The BL montage results in current loss through the inferior regions and the consideration of a limited FOV model misses accounting for this detail (see coronal images in Figure 3). As a result, the percentage of the brain volume stimulated above typical thresholds (neural activation and robust neural activation) for the RUL montage is lower than the BL montage—only for the upper head model. When the full-head and additional FOV models are considered, RUL exceeds the BL montage in the percentage of brain volume stimulated.

There is no debate that RUL ECT is considered cognitive sparing (7). Our finding that RUL stimulates the brain more than BL ECT presumably means that the focality metric as currently used is not indicative or predictive of the brain-sparing aspects of RUL ECT. Since focality is related to E_th_, one could speculate that the assumed E_th_ value may not be adequate. These threshold values were derived based on recorded E-field waveforms in combination with the neuronal time constant and E-field activation threshold data from prior TMS studies (9) and as such subject to multiple assumptions. Furthermore, seizure as opposed to the motor threshold may be more relevant to ECT investigations. Another possibility is the particular head shape contributing to the increased distance between the vertex and the temple electrodes, resulting in more current flow into the brain. The dataset considered is an average Caucasian dataset (MNI 152) and Caucasians in general have a more elongated shaped head than other races (24). However, ultimately, therapeutic seizures are induced when stimulus intensity exceeds seizure thresholds and there is no one-to-one correlation to the percentage of brain volume stimulated. Nonetheless, the E-field is meaningful as a lower value in a certain region (subthreshold) would mean a low probability of seizure initiation at that location. A third possibility is that the laterality of stimulation (Figure 5) is a more important metric than focality with respect to improved cognitive performance associated with RUL (30, 31). Our results demonstrate whole-brain stimulation relative to E_th_ with RUL and near whole-brain stimulation (~90%) with BL at 800 mA amplitudes (Figure 6). Previous ECT E-field investigations have demonstrated the E-field laterality associated with RUL (14, 32) and the association of diminished cognitive performance with higher E-fields (i.e., >112.5 V/m) (14). The RUL E-field in the left hemisphere is above E_th_ but may be insufficient to adversely impact cognition. Alternative thresholds (i.e., seizure threshold) may be useful to compare the focality differences associated with ECT electrode placements at amplitudes used in clinical practice (800 or 900 mA). Applying a higher threshold to the brain saturation maps would minimize the saturation effect of higher amplitudes and offer insights into electrode placement focality differences associated with higher E-fields.

While our study did not study other ECT placements such as bifrontal (BF) (25), it is possible to draw general conclusions based on the results of our study. As the dominant current flow of the BF montage is expected to flow in the superior regions (i.e., under and between the electrodes), we can fully expect lesser impact due to the modeled FOV in comparison to the RUL and the BL placements. In this case, even considering a FOV extending to the upper head may be sufficient. Since we used a single head model, we cannot account for inter-individual differences in calculated stimulation and focality metrics. We expect inter-individual differences as the prior effort involving a full-head extent model (3) indicated a greater percentage of brain volume stimulation for BL than RUL with the respective curves only merging at a high ECT amplitude of ~800 mA). Future efforts should, therefore, explore RUL- and BL-induced brain volume stimulation systematically across multiple individualized datasets.

## Data availability statement

The original contributions presented in the study are included in the article/supplementary material, further inquiries can be directed to the corresponding author.

## Ethics statement

Ethical review and approval was not required in accordance with institutional guidelines.

## Author contributions

AD developed the concept idea. AG performed the E-field modeling and related post-processing. AG and AD analyzed the results and prepared the initial manuscript draft with important intellectual input from CA, Z-DD, YH, PP-F, and DT. All authors confirmed the overall methodology, contributed to the final manuscript text, and approved the submitted version.
